# Zebrafish as an alternative animal model in human and animal vaccination research

**DOI:** 10.1186/s42826-020-00042-4

**Published:** 2020-05-07

**Authors:** Ricardo Lacava Bailone, Hirla Costa Silva Fukushima, Bianca Helena Ventura Fernandes, Luís Kluwe De Aguiar, Tatiana Corrêa, Helena Janke, Princia Grejo Setti, Roberto De Oliveira Roça, Ricardo Carneiro Borra

**Affiliations:** 1 Ministry of Agriculture, Livestock and Supply, Federal Inspection Service, São Carlos, SP Brazil; 2 São Paulo State University, Botucatu, SP Brazil; 3 Health and Biological Sciences Center, Federal University, Federal University of São Carlos, São Carlos, SP Brazil; 4 Technical Directorate for Teaching and Research Support, São Paulo University, São Paulo, SP Brazil; 5 Department of Food Technology and Innovation, Harper Adams University, Newport, UK; 6 Department of Genetic and Evolution, Federal University of São Carlos, São Carlos, SP Brazil

**Keywords:** 3Rs, Animal health, Human health, Immunity, Toxicology, Vaccine safety

## Abstract

Much of medical research relies on animal models to deepen knowledge of the causes of animal and human diseases, as well as to enable the development of innovative therapies. Despite rodents being the most widely used research model worldwide, in recent decades, the use of the zebrafish (*Danio rerio*) model has exponentially been adopted among the scientific community. This is because such a small tropical freshwater teleost fish has crucial genetic, anatomical and physiological homology with mammals. Therefore, zebrafish constitutes an excellent experimental model for behavioral, genetic and toxicological studies which unravels the mechanism of various human diseases. Furthermore, it serves well to test new therapeutic agents, such as the safety of new vaccines. The aim of this review was to provide a systematic literature review on the most recent studies carried out on the topic. It presents numerous advantages of this type of animal model in tests of efficacy and safety of both animal and human vaccines, thus highlighting gains in time and cost reduction of research and analyzes.

## Introduction

The role of the immune system is to protect a body against bacterial, viral, or any foreign antigen invasions. In order to improve protection, vaccination is used to boost immunity against diseases caused by microorganisms. It typically contains a less virulent agent that triggers a reaction, thus, stimulating a body’s immune system to recognize it as foreign. In the process, a body’s defense mechanism learns to recognize and destroy a microorganism, its toxins or surface proteins [94] every time an invasion is identified. The use of vaccination is important because it promotes the stimulation of the body’s defense mechanisms and the development of both individual and collective immunity. Vaccination can act on specific (adaptive) and nonspecific (innate) immune responses unlike immunostimulants which only act on innate response. In addition, it should be noted the role vaccines play in controlling diseases as preventative as well as non-therapeutic measures. Therefore, the body is able to produce antibodies that recognize, signal and neutralize pathogens or particular cellular responses which detect the specific antigens with high efficiency and affinity. As a result, vaccines protect the body against future infections [27] thus reducing the need for the use of antibiotics and other types of drugs.

Despite the study of immunology in fish being more recent compared to those of humans and in animals, the concepts and techniques used are similar [60]. The study of the use of vaccines in fish is an area of fast-growing. As aquaculture expands and the need to control pathogens becomes more pressing, the commercial vaccination of different varieties of fish is already a reality in many countries. It aids in the prevention of diseases that could pose health risks to the shoal as well as in avoiding the economic losses due to mortality caused by infection. It reduces the contamination of water bodies by the excessive use of antibiotics, and the reduction of final fish product quality [5, 24, 42, 79, 100].

The Zebrafish model has been widely used in both animal and human health research and, more recently, in aquaculture too. In spite of rodents being the most widely used research model in the world, in recent decades the use of the zebrafish (*Danio rerio*) model has exponentially increased among the scientific community. It follows the principle of 3Rs (replacement, reduction, and refinement) as required by a multiplicity of national and international regulatory bodies. Furthermore, the use of zebrafish model results in a reduction of time and use of resources when compared to those more established animals’ models. It also provides a greater informational and predictive capacity when compared to in vitro results [53]. Thus, using the zebrafish model, it is possible to replace and reduce the use of mammals in research as well as mitigate problems related to the welfare of those animals. Furthermore, zebrafish is used as confirmatory models of the positive previously obtained results, thus, having the ability to refine the findings [2]. A review of the literature was carried out aiming at presenting the most recent information on vaccination of fish, which brings to light the advantages of this animal model in tests of efficacy and safety of both animal and human vaccines.

## Material and methods

The present study was based on a systematic literature review carried out using databases such as Science Direct, Google Scholar and SciELO (Scientific Electronic Library Online). Emphasis was given on identifying publications using search words and terms containing ‘human vaccination’ and ‘animal vaccination’. Particularly, the main key-words searched included ‘Zebrafish model’, ‘vaccine safety’, ‘diseases’, ‘infection’ and ‘toxicology’. Initially, 99 publications were identified which included books, rulings and articles published by international scientific journals of high impact factor. The publications were selected according to relevance and timeliness. 19% of the articles used were published in the last year, 65% in the last 5 years, and 89% published in the last 10 years.

## Discussion

### Zebrafish model and vaccines testing

#### Vaccination safety

When devising immunization experiments, challenge trials for vaccine development evaluate the efficacy and safety of the vaccine against different pathogens. These are normally assessed using animal models, mainly mammals, which are often imprecise in reflecting human diseases [93], not to mention time consuming, and require a large number of animals. Moreover, the mortality and clinical signs as well as laboratory tests are usually analyzed to evaluate the innate (non-specific) or adaptive (specific) immune system response. As in mammals, Zebrafish has a well-maintained adaptive immune system composed of T and B lymphocytes that develop from the thymus and kidneys respectively. However, in relation to the development of memory lymphocytes, fish seem to have memory cells of the type B and T [78]. Yet, there has not been enough data to confirm that in Zebrafish. Zebrafish also presents the enzyme system involved in the process of genetic rearrangement that originates the B (BCR) and T (TCR) lymphocyte receptors. As in humans, Zebrafish has recombination activator genes that control the rearrangement of gene segments V, D and J to produce the diversity of antibodies and lymphocyte receptors. In addition, the zebrafish’s immune system has only approximately 300,000 antibody-producing B cells, making it three orders of magnitude smaller than mice and five orders simpler than humans [48].

The efficiency of the humoral response increases due to the increased affinity of the antibodies. Affinity maturation of antibody responses is less efficient in cold-blooded vertebrates compared to mammals. Despite this, in zebrafish, data revealed that specific nucleotides in regions of the BCR receptor were target of directed mutations. Therefore it was suggested that activation-induced deaminase and affinity maturation contributed to the diversification of antibodies also in fish [56]. Immunization of teleost fish with the TNP-KLH antigen (linked to trinitrophenyl to keyhole limpet hemocyanin), for example, induced the production of specific low affinity antibodies, which were replaced in 5 weeks by antibodies of intermediate affinity, and after 15 weeks, by antibodies with greater affinity for the antigen [28, 97].

Among the immunological tests, the most frequent ones are: complete hematological analysis by counting erythrocytes; thrombocytes and leukocytes; differential white cell count; hematocrit; glucose; organ histology, and immunological essays such as serology, specific antibody titration, and agglutination [4, 29, 57]. Furthermore, toxicity tests can be also conducted using zebrafish such as embryotoxicity, hepatotoxicity, neurotoxicity, endocrine toxicity, genotoxicity, among others as proposed by Bailone et al. [3].

**Fig. 1:**
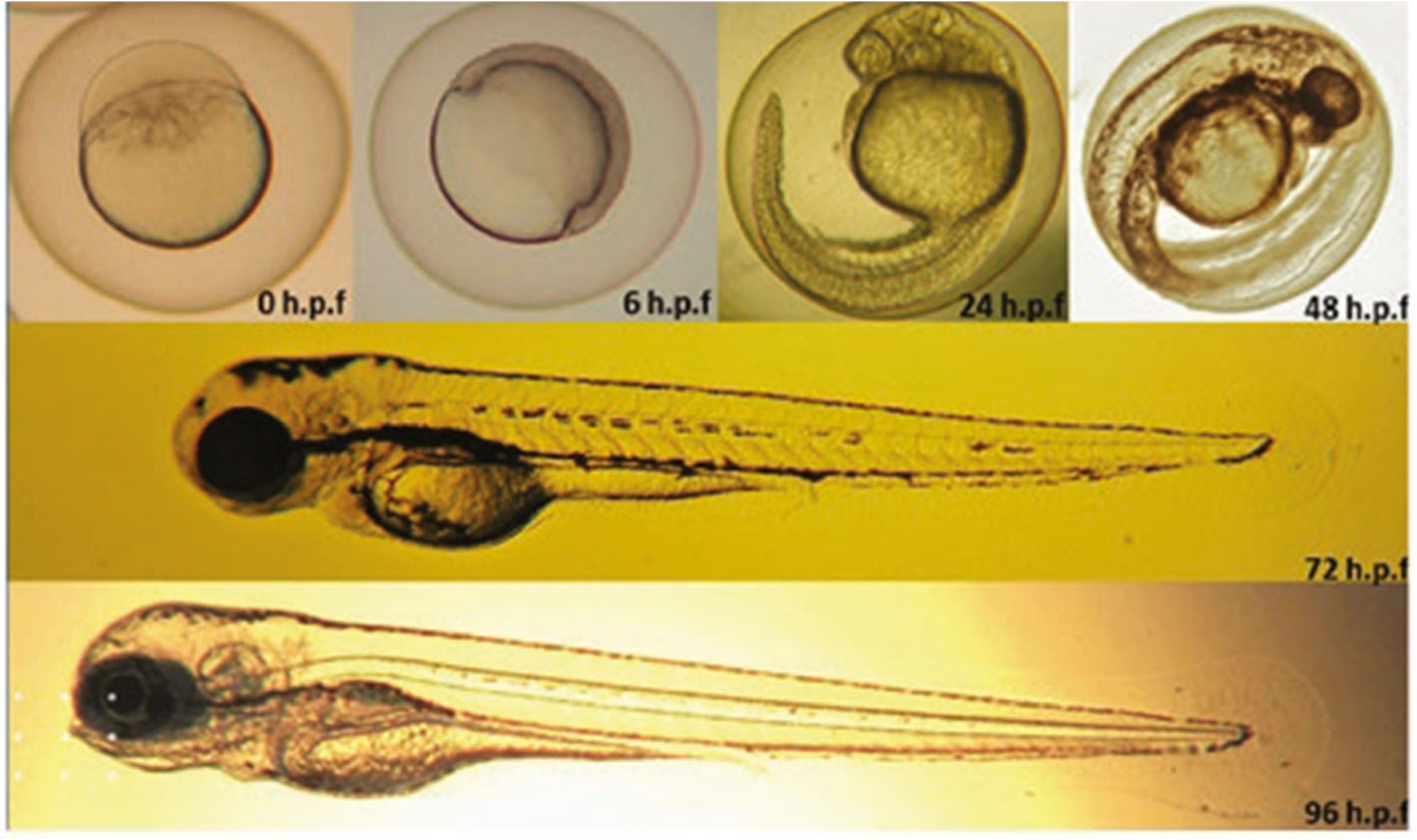
Embryos of zebrafish 0, 6, 24 and 48 h’ post-fertilization. Larvae of zebrafish 72 and 96 h post-fertilization

Up to now, these tests have been conducted using rodents, but in recent decades, the Zebrafish model has proved to be an important tool in the studies of infections and immunological responses. This model has the advantage of having OECD-specific guidelines for safety evaluation of chemical compounds (acute toxicity), which is performed within 96 h [65]. In addition, observations can be made in real-time allowing for the monitoring of embryogenesis (Fig. 1) as well as regarding the effects of vaccines in relation to cardiovascular, hepatic, nervous, and endocrine, not to mention, behavioral aspects too [18, 40].

Prior to vaccines being tested on humans, livestock or pets, these should be assessed using animal models to avoid causing them harm, including death, especially in the case of immunosuppressed organisms, children and the elderly [26]. As for vaccination in humans, for example, about 0.4 to 1.9 people per million who had been vaccinated with BCG against tuberculosis may have developed the disease through vaccine contagion. For hepatitis B, 1 in 600,000 people vaccinated may have presented a severe allergic reaction (anaphylaxis). In the case of vaccine against poliomyelitis, vaccine contagion happened to 1 in every 3.6 million vaccinated. Moreover, to combat yellow fever, the vaccine contagion and seizures happened to 1 in 22 million and internal hemorrhages happened to 1 in 450,000. Thence, the occurrence of side effects is very rare. Side effect reactions in humans may also be observed to be caused by other vaccines such as yellow fever, measles, mumps, rubella, chicken pox, diphtheria and tetanus. The most common symptoms are seizures, severe allergic reactions, meningitis, encephalitis [26]. Although these risks are irrelevant when compared to damages that could be caused by the non-use of a vaccine, the toxicology, the side effects and immunization at different concentrations ought to be adequately tested.

**Fig. 2:**
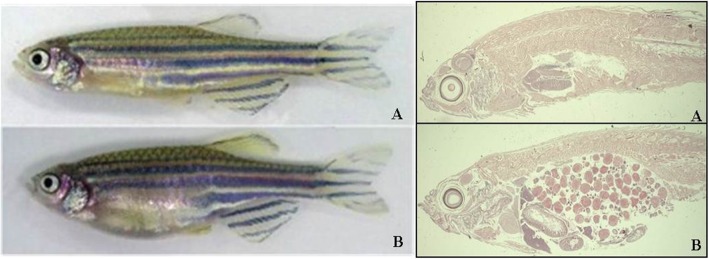
Histology of adult zebrafish (hematoxylin eosin). **a** Male. **b** Female

Thus, the Zebrafish model has the advantage of a researcher to follow in real-time the fish’s development from its embryogenesis to full organ development which is reached about 36 h after fertilization. This allows for a vaccine’s effect on all the major organs precursors to be closely studied [53] such as using immunohistology (Fig. 2).

Zebrafish and mammalian toxicity (Lethal concentration – LC_50_) profiles are surprisingly similar for a range of substances specified in Table 1 below. Therefore, toxicity studies support the effectiveness of using the zebrafish model for the purpose of testing these substances. Furthermore, they can be extrapolated to the active ingredients present in the vaccine, and enabling quick parallel studies of vaccine reactions in humans and zebrafish.

**Table 1:** Range of drugs used in human medicine with similar results of toxicity (LC_50_) in zebrafish

Drug	Used as
Geladanamycin	Antibiotic
Ethanol (Ethyl alcohol)	Antiseptic
Dexamethasone (Corticosteroid)	Anti-inflammatory
Acetaminophen	Analgesic/ antipyretic
Doxorubicin	Used in cancer chemotherapy
Aspirin (Acetylsalicylic acid)	Analgesic / antipyretic / anti-inflammatory
Amiodarone	Antiarrhythmic / potent vasodilator
Tacrine	Reversible acetylcholinesterase inhibitor
Cyclosporine A	Immunosupressor drug
Didemnin B	Antiviral / immunosuppressor

Source: Adapted from Kari et al. [43] *apud* Zhang et al. [99]

#### Advantages of zebrafish model in vaccination tests

Compared to other vertebrates, zebrafish have extra biological advantages including high fecundity, external fertilization, optical transparency and rapid development. Moreover, Zebrafish possess a highly developed immune system that is remarkably similar to the human one. Therefore, it is expected that the majority of the signaling pathways and molecules involved in the immune response of mammals would also exist and behave similarly in fish [89]. Consequently, the presence in fish of elements of innate and adaptive immunity enables research in infectious processes, being susceptible to infections by gram-negative and gram-positive bacteria, protozoa, viruses, fungi and mycobacteria.

The development of special cloning, mutagenesis and transgenesis techniques allowed the identification of a significant number of mutants. Commercial mutant zebrafish lines and the recently developed CRISPR/Cas9 genome modification system provide the means to create knockout zebrafish for studying individual genes at a whole organism level [66]. Non-pigmenting mutants such as Casper zebrafish have also helped improve visibility of internal organs [92]. In addition it is easy to generate transgenic zebrafish with ‘reporter genes’ to facilitate analysis in live fish [87]. Because the zebrafish genome is conserved in humans, information obtained from zebrafish studies may lead to translational results in humans [38].

Examples of mutant animals displaying human-like diseases are numerous such as: *sapje,* which has the gene homologous to that of Duchenne muscular dystrophy; *dracula*, related to erythropoietic protoporphyria; *van Gogh,* model of the DiGeorge syndrome; and *gridlock*, which induces coarctation of the aorta [47]. Research in tumor suppressor genes *p53* and *apc* (*adenomatous polyposis coli)* is another area of great interest*.* The importance of the *p53* gene in human carcinogenesis is well recognized and recent studies have shown zebrafish as an excellent model for assessing the presence (or not) of gene stability. Lymphoid leukemia, melanoma and hepato-carcinoma have already been described in zebrafish thus confirming that the molecular mechanisms involved are similar to those of humans [49].

**Fig. 3:**
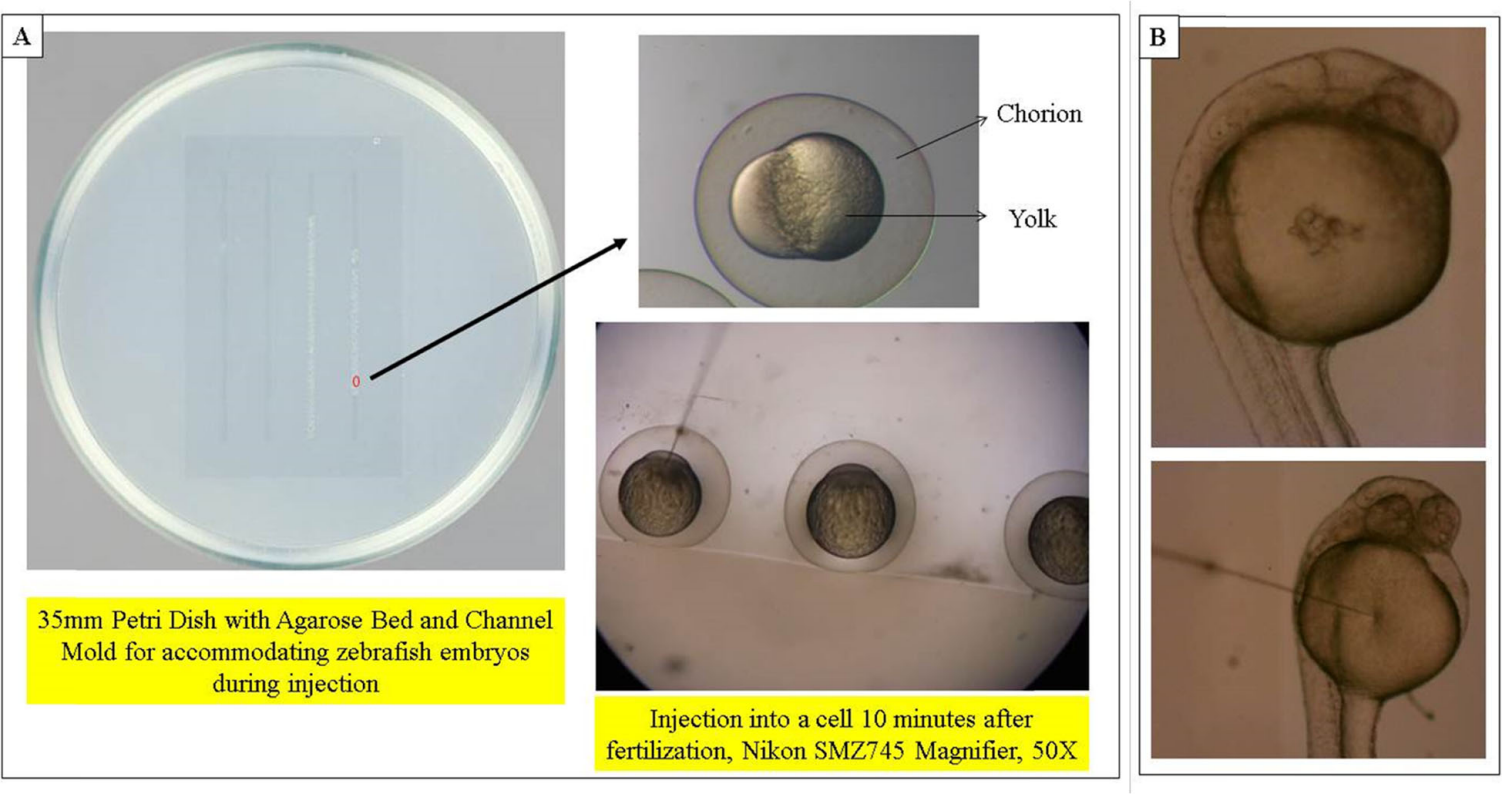
**a** Vitelline Yolk Injection (24 HPF), Magnifying Glass Nikon SMZ745, 50X; B) Vitelline Yolk Injection (24 h.p.f.), Magnifying Glass Nikon SMZ745, 50X

**Fig. 4:**
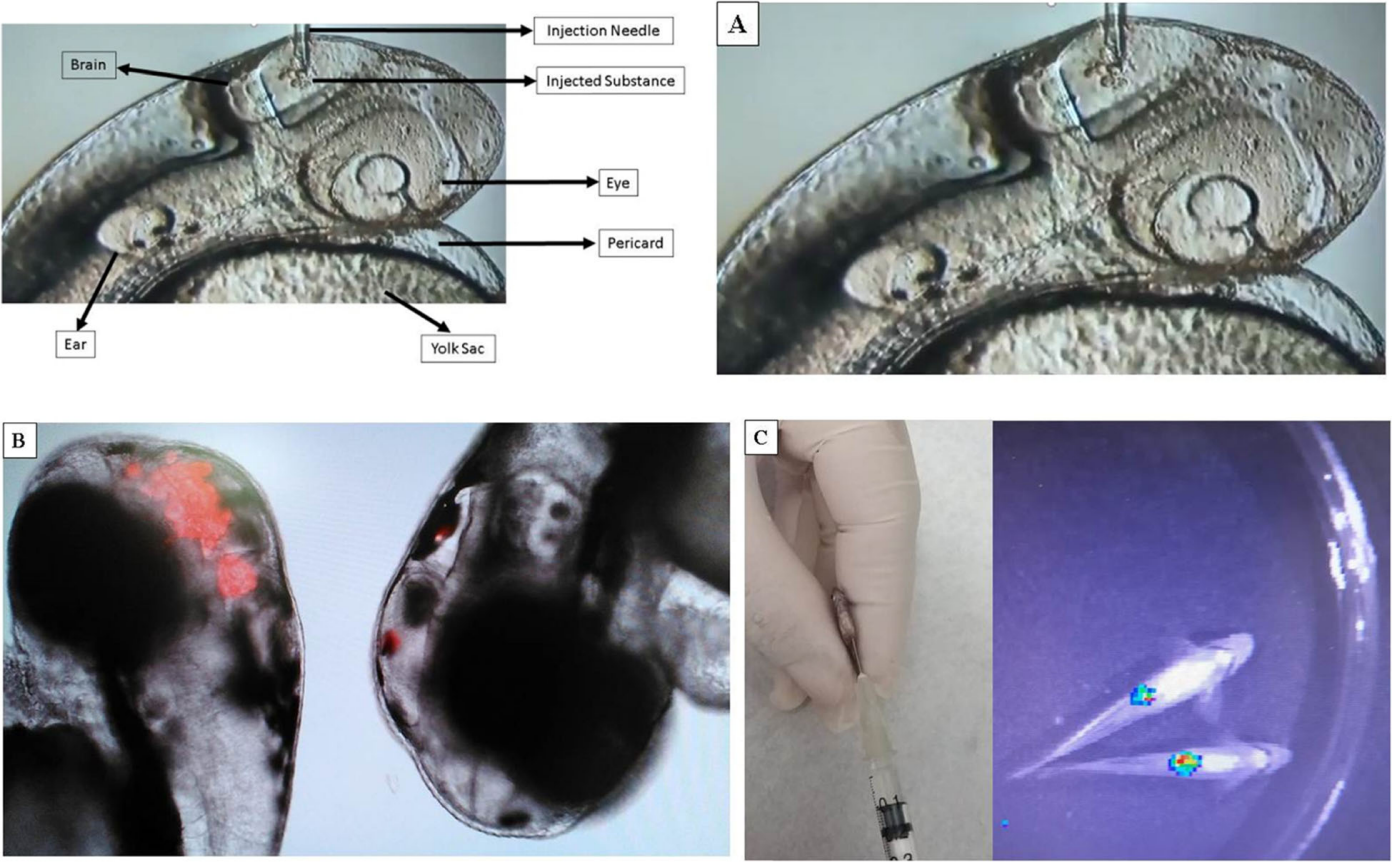
**a** 24 HPF Zebrafish Embryo Brain Injection, Nikon Microscope; **b** Brain injection of turbo-red substance into a 24 HPF zebrafish embryo; **c** Luciferin-labeled 4 T1 tumor cell bioluminescence in 3-month-old animals

Regarding the administration of vaccines, in view of the different routes of applications presented in animals and humans, the zebrafish model still allows the immunization of embryos, facilitated by its transparency, using glass needles (Figs. 3 and 4). Interestingly, the fact that the fish’s adaptive immune system does not reach maturity up to 4 weeks after fertilization allows them to be used without the need for immunosuppression in the embryonic stages [32] in the case, for example, of tumor xenograft experiments.

In zebrafish larvae, a rapid systemic infection can be initiated by direct microinjection of a bacterial suspension into the bloodstream. Alternatively, a more localized infection may be induced by the injection of microbes into the muscle tail or the hindbrain ventricle [6]. For high transfer rate, the microbes can be readily injected into the yolk for the first few hours after fertilization. However, it is important to keep in mind that the yolk lacks immune cells, and therefore the bacteria are able to grow freely before invading the larval tissues [51].

Several transgenic zebrafish lines containing fluorescent markers in different cells of the immune system have been developed to visualize host-microbe interactions in the transparent larvae. For example, recruitment of fluorescent neutrophils to the site of bacterial infection (which can also be labeled with fluorescence) could be easily followed and quantified in real time. Yet, so far, researchers have focused primarily on larval infection patterns [51].

#### Fish vaccines

In the prevention of disease outbreaks causing mortalities in aquaculture, similarly to any other animal production system, vaccination is essential. Thus, the use of vaccines for that purpose could be improved based on the results from the studies performed in zebrafish [89]. The development of vaccines for aquaculture has been an important milestone for guaranteeing a continuous safe and high standard animal health production system. In recent years, zebrafish models have been chosen as the preferred model in the production of fish vaccination experiments against several pathogens that cause losses in aquaculture around the world such as bacteriosis and viruses. One of the most important pathogen studies applied to fishing production is attributed to Guo et al. [35]. They analyzed the protective efficacy of four iron-related recombinant proteins and their single-walled carbon nanotube encapsulated counterparts against the *Aeromonas hydrophila* infection in zebrafish. They observed that the immune response was increased after vaccination. Guo et al. [34] also studied *Edwardsiella tarda* which is an important intracellular pathogenic bacterium that causes the infectious disease Edwardsiellosis in fish. They proved that live *E. tarda* vaccine enhanced innate immunity by metabolic modulation in zebrafish.

*Vibrio anguillarum*, a bacterium that causes vibriosis, was also studied by Ye et al. [98] who observed the maternal transfer and protection role in zebrafish offspring following vaccination of the brood stock with a live attenuated *V. anguillarum* vaccine. They proved that the development of immune cells was enhanced and the maternally-derived antibody could protect early embryos and larvae from the attack of specific pathogens via vaccination with a live attenuated vaccine. Furthermore, Liu et al. [50] analyzed the profiling immune response in zebrafish intestine, skin, spleen and kidney when immersion vaccinated was used with a live attenuated V. anguillarum vaccine. Immersion, or bath vaccination, is a common practice in aquaculture, because of it being convenient as mass vaccination giving sufficient protection. The fish is submerged in water with a sub lethal concentration of the bacteria for a specific time. Liu et al. [50] observed that antibodies were either produced at antigen-contact tissues or in immune organs. Zhang et al. [101] studied Th17-like immune response in fish mucosal tissues after administration of live attenuated *V. anguillarum* via different vaccination routes. When compared to injection vaccination, immersion vaccination elicited intense Th17-like immune responses in the gut tissue of zebrafish. *Vibrio vulnificus*, that is an aquatic pathogen that can cause primary sepsis and soft tissue infection, was also tested during an experimentation of zebrafish’s reaction to vaccine. It was concluded that CpG oligodeoxynucleotides, a type of essential immunomodulators, protected zebrafish against *Vibrio vulnificus* induced infection [15].

*Francisella noatunensis* is a bacterium that causes granulomatous disease in freshwater and marine fish, and remains an unsolved problem for the aquaculture sector as no efficient vaccines are yet available. Lagos et al. [46] studied the immunomodulatory properties of *Concholepas concholepas* hemocyanin against francisellosis in a zebrafish model, proving that his adjuvant was a potential one for aquaculture vaccines. Moreover, Brudal et al. [11] observed that vaccination with outer membrane vesicles from *F. noatunensis* reduced the development of francisellosis in a zebrafish model.

Streptococcus sp. has also been studied with the Zebrafish model. *Streptococcus parauberis* is the major infectious agent of streptococcosis in olive flounder (*Paralichthys olivaceus*). Kim et al. [45], studying the identification of novel immunogenic proteins against *S. parauberis* by reverse vaccinology using zebrafish model, identified 41 vaccine candidates against *S. parauberis.* Furthermore, *Streptococcus iniae* was studied by Membrebe et al. [58] testing the protective efficacy of *Streptococcus iniae* derived enolase against Streptococcal infection in zebrafish model. In that study, enolase protein was evaluated to induce cross-protective immunity against *S. iniae* and *S. parauberis* which are major pathogens causing streptococcosis in fish.

Further to the aforementioned examples, many other diseases have been investigated with the Zebrafish model. For example, Rhabdovirus, which is one of the most important diseases in salmonids, is a virus that causes hemorrhagic viral septicemia [44, 64]. *Listeria monocytogenes* [19, 20]; *Piscirickettsia salmonis* which causes salmonid rickettsia sepsis (Tandberg et al. [83]); and in adjuvant test to improve the efficacy of vaccines [44], among others [82].

#### Animals and human vaccines

The zebrafish model has been used not only in aquaculture, but also in veterinary and human medicine. So far, it has become one of the major model systems used in modern biomedical research [51]. According to Torraca et al. [86], zebrafish can be also used as a model for pathogenesis and host defense, modeling many human diseases, such as tuberculosis, *Staphylococcus aureus* and Shigella infection, among others, as well as model to investigate immune cells, infection and inflammation of different kind of human diseases.

Torraca et al. [86] posited that zebrafish could also be used as a model for *Tuberculosis* which is a devastating infectious disease worldwide and with no current prospect of efficient prevention. Tuberculosis is an infectious disease caused by bacilli from the *Mycobacterium tuberculosis* complex. It is estimated that up to one third of the world’s population is infected with *M. tuberculosis* and have active tuberculosis, which often develops decades after the primary infection. Annually about two million people perish of tuberculosis and, so far, due to the lack of well-established animal models, such a disease has been difficult to study [51].

An infection by *Mycobacterium marinum* in adult zebrafish resembles that of human tuberculosis, as demonstrated by Myllymäki et al. [62]. Those authors proved that the *M. marinum* infection model in adult zebrafish was suitable for preclinical screening of tuberculosis immune’s responses and vaccines. It was also a promising new model for tuberculosis vaccine research, including the pre-clinical identification of vaccine antigens [16, 17, 36, 41, 61, 67];). Other species of *Mycobacterium* have also been studied, such as *M. bovis* [52, 73] and *M. abscessos* [7]. *M. bovis* is most common in cattle, but also affects humans. *M. bovis* Bacillus Calmette-Guérin vaccine is currently available as a prophylactic tool for preventing the disease. It has been shown to be efficient in preventing disseminated forms of tuberculosis in children; however, its efficiency is limited in areas where individuals have had prior exposure to environmental mycobacteria, and its efficacy decreased with a host’s age [55].

Moreover, teleost models offer an expanding platform for the understanding of mycobacterial infections and those mechanisms that offer the greatest potential to enhance host protection [37]. The models make it possible to screen the host and bacterial factors that modify the disease and facilitate the search for new therapeutic agents. It has recently been shown that zebrafish can also be used for the potential screening of DNA-based vaccines and, in particular, for identifying novel antigens protecting against mycobacteria [67]. Therefore, using the Zebrafish model is expected to accelerate the understanding of the pathogenesis of tuberculosis which would lead to the development of better vaccines. Yet, the usefulness of this model is not limited to tuberculosis, which as seen before it could benefit research for many other important infectious diseases [51].

Similarly, this model also helps to elucidate bacterial infections in animals and humans by *Aeromonas hydrophila* [91], *Pseudomonas aeruginosa* [74], *Escherichia coli* nonpathogenic [63], *E. coli* CFT073 [95], *Listeria monocytogenes* [80, 81], *Myroides odoratimimus* [72], *Cronobacter turicensis* [25], *Streptococcus agalactiae* [70, 96],  *Streptococcus iniae* and *Streptococcus pyogenes* [59, 76, 77], among others [12, 85].

Shigella is a major cause of dysentery worldwide, accounting for up to 165 million cases of shigellosis each year [23]. Yet, despite there not existing vaccine available as yet, the human and animal challenge–rechallenge trials with virulent Shigella as well as observational studies in Shigella-endemic areas are promising. The incidence of the disease decreased following Shigella’s infection which pointsto a biological feasibility of a vaccine [54]. Phalipon et al. [71] as well as Mani et al. [54] proposed that adult zebrafish could be used to study the immune response to Shigella, which is crucial to understanding the crosstalk between Shigella and T-lymphocytes [75] thus this being relevant in the development of vaccine strategies. Studies have also been conducted with Zebrafish model to promote a vaccine against Salmonella, which produces gastroenteritis that causes massive morbidity and mortality in adults and children in developing countries. Howlader et al. [39] proved that zebrafish was an excellent model for the study of vaccines using successive immersion triple vaccines with the single serotype *Salmonella. Typhimurium* and *Salmonella entereditis* induced protective efficacy against a high dose (10^8^ CFU/ml) of infection by these pathogens.

Other microorganisms of importance such as fungi which can cause pathologies in humans, such as *Candida albicans* [10], *Cryptococcus neoformans* [8, 84] and *Mucor circinelloides* [90] have also been the subject of study with teleosts. In addition, viruses such as Herpes simplex [13, 31]; human norovirus [88]; Vesicular stomatitis [33]; hepatite C [21, 22]; Chikungunya [1, 9, 14, 68]; Sindib [69] and Influenza A [30] are some of the human viruses already studied by the zebrafish model in both embryos and larvae.

## Conclusions

The use of the Zebrafish model for the production of vaccines with application for both animals and humans, despite already being a reality, is still underused. This model is an important tool for the development of new safe vaccines against diseases which do not yet have preventive treatment, or for which the existing vaccines are not so effective. Thus, previous screening tests with zebrafish have been proven to be effective in preliminary phases prior to testing with mammalians. Despite the evidence from the literature indicating that science in this field is in its infancy, when compared to other animal models used in research, teleost models have proved to be effective in the elucidation of the infection and immunological responses to the diverse animal and human pathogens. In addition, the reduced financial cost and time frame needed for testing are another attractive regarding the use of zebrafish. Thus, it is expected its use would expand in the coming years.

## Data Availability

See Materials and Methods section;

## References

[1] Aleksejeva E, Houel A, Briolat V, Levraud JP, Langevin C, Boudinot P (2016). Zebrafish Plzf transcription factors enhance early type I IFN response induced by two non-enveloped RNA viruses. Dev Comp Immunol.

[2] Bailone RL, Aguiar LK, Roça RO, Borra RC, Corrêa T, Janke H (2019). Zebrafish as an animal model for food safety research: trends in the animal research. Food Biotechnol.

[3] Bailone RL, Fukushima H, Roça R, Corrêa T, Janke H, Setti P, et al. Potenciais usos do Modelo animal zebrafish *Danio rerio* em pesquisas na medicina veterinária. Rev Educ Cont Med Vet e Zoo CRMV-SP. 2019b; Just Accepted.

[4] Bailone RL, Martins ML, Mouriño JLP, Vieira FN, Pedrotti FS, Nunes GC (2010). Hematology and agglutination titer after polyvalent immunization and subsequent challenge with *Aeromonas hydrophila* in Nile tilapia (*Oreochromis niloticus*). Arch Med Vet.

[5] Bao P, Sun X, Liu Q, Zhang Y, Liu X (2019). Synergistic effect of a combined live *Vibrio anguillarum* and *Edwardsiella piscicida* vaccine in turbot. Fish Shellfish Immunol.

[6] Benard EL, Van der Sar AM, Ellett F, Lieschke GJ, Spaink HP, Meijer AH (2012). Infection of zebrafish embryos with intracellular bacterial pathogens. JoVE (J Vis Exp).

[7] Bernut A, Dupont C, Sahuquet A, Herrmann JL, Lutfalla G, Kremer L (2015). Deciphering and imaging pathogenesis and cording of *Mycobacterium abscessus* in zebrafish embryos. JoVE (J Vis Exp).

[8] Bojarczuk A, Miller KA, Hotham R, Lewis A, Ogryzko NV, Kamuyango AA (2016). *Cryptococcus neoformans* intracellular proliferation and capsule size determines early macrophage control of infection. Sci Rep.

[9] Briolat V, Jouneau L, Carvalho R, Palha N, Langevin C, Herbomel P (2014). Contrasted innate responses to two viruses in zebrafish: insights into the ancestral repertoire of vertebrate IFN-stimulated genes. J Immunol.

[10] Brothers KM, Newman ZR, Wheeler RT (2011). Live imaging of disseminated candidiasis in zebrafish reveals role of phagocyte oxidase in limiting filamentous growth. Eukaryot Cell.

[11] Brudal E, Lampe EO, Reubsaet L, Roos N, Hegna IK, Thrane IM (2015). Vaccination with outer membrane vesicles from *Francisella noatunensis* reduces development of francisellosis in a zebrafish model. Fish Shellfish Immunol.

[12] Brudal E, Ulanova LS, Lampe EO, Rishovd AL, Griffiths G, Winther-Larsen HC (2014). Establishment of three Francisella infections in zebrafish embryos at different temperatures. Infect Immun.

[13] Burgos JS, Ripoll-Gomez J, Alfaro JM, Sastre I, Valdivieso F (2008). Zebrafish as a new model for herpes simplex virus type 1 infection. Zebrafish..

[14] Burnham LA, Jaishankar D, Thompson JM, Jones KS, Shukla D, Tiwari V (2016). Liposome-mediated herpes simplex virus uptake is glycoprotein-D receptor-independent but requires heparan sulfate. Front Microbiol.

[15] Chen H, Zhang L, Li S, Ling K, Chen X, Lin C. CpG-ODN 2007 protects zebrafish against *Vibrio vulnificus*-induced infection. bioRxiv. 2019:780742. 10.1101/780742.

[16] Cen J, Jia ZL, Zhu CY, Wang XF, Zhang F, Chen WY, Liu KC, Li, SY, Zhang, Y. Particulate matter (PM10) induces cardiovascular developmental toxicity in zebrafish embryos and larvae via the ERS, Nrf2 and Wnt pathways. Chemosphere. 2020. p. 126288. 10.1016/j.chemosphere.2020.126288 32114347

[17] Cheng T, Kam JY, Johansen MD, Oehlers SH (2020). High content analysis of granuloma histology and neutrophilic inflammation in adult zebrafish infected with *Mycobacterium marinum*. Micron..

[18] Cornet C, Calzolari S, Miñana-Prieto R, Dyballa S, Van Doornmalen E, Rutjes H (2017). ZeGlobalTox: an innovative approach to address organ drug toxicity using zebrafish. Int J Mol Sci.

[19] Ding C, Fan E, Wang S, Guo L, Li J, Liu Q (2017). A potential aquaculture vaccine vector: evaluation of a double-gene attenuated *Listeria monocytogenes* in zebrafish (*Danio rerio*). Aquaculture..

[20] Ding C, Liu Q, Li J, Ma J, Wang S, Dong Q (2019). Attenuated *Listeria monocytogenes* protecting zebrafish (*Danio rerio*) against vibrio species challenge. Microb Pathog.

[21] Ding CB, Zhang JP, Zhao Y, Peng ZG, Song DQ, Jiang JD (2011). Zebrafish as a potential model organism for drug test against hepatitis C virus. PLoS One.

[22] Ding CB, Zhao Y, Zhang JP, Peng ZG, Song DQ, Jiang JD (2015). A zebrafish model for subgenomic hepatitis C virus replication. Int J Mol Med.

[23] Duggan GM, Mostowy S (2018). Use of zebrafish to study Shigella infection. Dis Model Mech.

[24] Faber MN, Holland JW, Secombes CJ (2019). Vaccination strategies and IgM responses against PKD in rainbow trout. Fish Shellfish Immunol.

[25] Fehr A, Eshwar AK, Neuhauss SC, Ruetten M, Lehner A, Vaughan L (2015). Evaluation of zebrafish as a model to study the pathogenesis of the opportunistic pathogen *Cronobacter turicensis*. Emerg Microbes Infect.

[26] Ferrairo F. Os riscos reais da vacina. Revista Superinteressante. 2015; https://super.abril.com.br/saude/os-riscos-reais-da-vacina/ Accessed 27 Feb 2020.

[27] Figueiredo HCP, Castro GAC, Leal CAG, Netto LN (2009). Uso de vacinas na piscicultura: verdades, mitos e perspectivas. Panorama da Aquicultura.

[28] Fillatreau S, Six A, Magadan S, Castro R, Sunyer JO, Boudinot P (2013). The astonishing diversity of Ig classes and B cell repertoires in teleost fish. Front Immunol.

[29] Fukushima HCS, Leal CAG, Cavalcante RB, Figueiredo HCP, Arijo S, Moriñigo MA (2017). *Lactococcus garvieae* outbreaks in Brazilian farms Lactococcosis in *Pseudoplatystoma* sp.–development of an autogenous vaccine as a control strategy. J Fish Dis.

[30] Gabor KA, Goody MF, Mowel WK, Breitbach ME, Gratacap RL, Witten PE (2014). Influenza a virus infection in zebrafish recapitulates mammalian infection and sensitivity to anti-influenza drug treatment. Dis Model Mech.

[31] Ge R, Zhou Y, Peng R, Wang R, Li M, Zhang Y (2015). Conservation of the STING-mediated cytosolic DNA sensing pathway in zebrafish. J Virol.

[32] Granato M, Nüsslein-Volhard C (1996). Fishing for genes controlling development. Curr Opin Genet Dev.

[33] Guerra-Varela J, Baz-Martinez M, Da Silva-Alvarez S, Losada AP, Quiroga MI, Collado M (2018). Susceptibility of zebrafish to vesicular stomatitis virus infection. Zebrafish..

[34] Guo C, Peng B, Song M, Wu CW, Yang MJ, Zhang JY (2015). Live *Edwardsiella tarda* vaccine enhances innate immunity by metabolic modulation in zebrafish. Fish Shellfish Immunol.

[35] Guo Z, Lin Y, Wang X, Fu Y, Lin W, Lin X (2018). The protective efficacy of four iron-related recombinant proteins and their single-walled carbon nanotube encapsulated counterparts against *Aeromonas hydrophila* infection in zebrafish. Fish Shellfish Immunol.

[36] Harjula SKE, Saralahti AK, Ojanen MJ, Rantapero T, Uusi-Mäkelä MI, Nykter M (2020). Characterization of immune response against *Mycobacterium marinum* infection in the main hematopoietic organ of adult zebrafish (*Danio rerio*). Dev Comp Immunol.

[37] Hodgkinson JW, Belosevic M, Elks PM, Barreda DR. Teleost contributions to the understanding of mycobacterial diseases. Dev Comp Immunol. 2019. 10.1016/j.dci.2019.02.011. 10.1016/j.dci.2019.02.011 30776420

[38] Howe K, Clark MD, Torroja CF, Torrance J, Berthelot C, Muffato M (2013). The zebrafish reference genome sequence and its relationship to the human genome. Nature..

[39] Howlader DR, Sinha R, Nag D, Majumder N, Mukherjee P, Bhaumik U (2016). Zebrafish as a novel model for non-typhoidal salmonella pathogenesis, transmission and vaccine efficacy. Vaccine..

[40] Jarque S, Ibarra J, Rubio-Brotons M, García-Fernández J, Terriente J (2019). Multiplex analysis platform for endocrine disruption prediction using zebrafish. Int J Mol Sci.

[41] Ji J, Torrealba D, Thwaite R, Gomez AC, Parra D, Roher N (2019). Nanostructured TNFα protein targets the zebrafish (*Danio rerio*) immune system through mucosal surfaces and improves the survival after *Mycobacterium marinum* lethal infection. Aquaculture..

[42] Kamalii A, Prabu E, Ruby P, Ahilan B (2018). Advanced developments in fish vaccination. J Aquacult Trop.

[43] Kari G, Rodeck U, Dicker AP (2007). Zebrafish: an emerging model system for human disease and drug discovery. Clin Pharm Ther.

[44] Kavaliauskis A, Arnemo M, Speth M, Lagos L, Rishovd AL, Estepa A (2016). Protective effect of a recombinant VHSV-G vaccine using poly (I: C) loaded nanoparticles as an adjuvant in zebrafish (*Danio rerio*) infection model. Dev Comp Immunol.

[45] Kim YS, Yoon NK, Karisa N, Seo SH, Lee JS, Yoo SS (2019). Identification of novel immunogenic proteins against *Streptococcus parauberis* in a zebrafish model by reverse vaccinology. Microb Pathog.

[46] Lagos L, Tandberg JI, Becker MI, Winther-Larsen HC (2017). Immunomodulatory properties of *Concholepas concholepas* hemocyanin against francisellosis in a zebrafish model. Fish Shellfish Immunol.

[47] Lieschke GJ, Currie PD (2007). Animal models of human disease: zebrafish swim into view. Nat Rev Genet.

[48] Litman GW, Cannon JP, Dishaw JL (2005). Reconstructing immune phylogeny: new perspectives. Nat Rev Immunol.

[49] Liu S, Steven DL. Zebrafish models for cancer. Ann Rev Pathol: Mech. 2011. 10.1146/annurev-pathol-011110-130330.

[50] Liu X, Wu H, Liu Q, Wang Q, Xiao J, Chang X (2015). Profiling immune response in zebrafish intestine, skin, spleen and kidney bath-vaccinated with a live attenuated *Vibrio anguillarum* vaccine. Fish Shellfish Immunol.

[51] Lohi O, Parikka M, Rämet M (2013). The zebrafish as a model for paediatric diseases. Acta Paediatr.

[52] López V, Risalde MA, Contreras M, Mateos-Hernández L, Vicente J, Gortázar C (2018). Heat-inactivated *Mycobacterium bovis* protects zebrafish against mycobacteriosis. J Fish Dis.

[53] MacRae CA, Peterson RT (2015). Zebrafish as tools for drug discovery. Nat Rev Drug Discov.

[54] Mani S, Wierzba T, Walker RI (2016). Status of vaccine research and development for Shigella. Vaccine..

[55] Mantilla Galindo A, Ocampo M, Patarroyo MA (2019). Experimental models used in evaluating anti-tuberculosis vaccines: the latest advances in the field. Expert Rev Vaccines.

[56] Marianes AE, Zimmerman AM (2011). Targets of somatic hypermutation within immunoglobulin light chain genes in zebrafish. Immunology..

[57] McFetridge R, Sobanjo-ter Meulen A, Folkerth SD, Hoekstra JA, Dallas M, Hoover PA (2015). Safety, tolerability, and immunogenicity of 15-valent pneumococcal conjugate vaccine in healthy adults. Vaccine..

[58] Membrebe JD, Yoon NK, Hong M, Lee J, Lee H, Park K (2016). Protective efficacy of *Streptococcus iniae* derived enolase against streptococcal infection in a zebrafish model. Vet Immunol Immunopathol.

[59] Miller JD, Neely MN (2004). Zebrafish as a model host for streptococcal pathogenesis. Acta Trop.

[60] Muktar Y, Tesfaye S, Tesfaye B (2016). Present status and future prospects of fish vaccination: a review. J Vet Sci Technol.

[61] Myllymäki H, Niskanen M, Luukinen H, Parikka M, Rämet M (2018). Identification of protective postexposure mycobacterial vaccine antigens using an immunosuppression-based reactivation model in the zebrafish. Dis Model Mech.

[62] Myllymäki H, Niskanen M, Oksanen KE, Sherwood E, Ahava M, Parikka M (2017). Identification of novel antigen candidates for a tuberculosis vaccine in the adult zebrafish (*Danio rerio*). PLoS One.

[63] Nguyen-Chi M, Phan QT, Gonzalez C, Dubremetz JF, Levraud JP, Lutfalla G (2014). Transient infection of the zebrafish notochord with *E. coli* induces chronic inflammation. Dis Model Mech.

[64] Novoa B, Romero A, Mulero V, Rodriguez I, Fernandez I, Figueras A (2006). Zebrafish (*Danio rerio*) as a model for the study of vaccination against viral haemorrhagic septicemia virus (VHSV). Vaccine..

[65] OECD (2013). Test no. 236: fish embryo acute toxicity OECD guidelines for the testing of chemicals.

[66] Ojanen M (2019). Reverse genetics to study immunity against mycobacteria in zebrafish (*Danio rerio*).

[67] Oksanen KE, Halfpenny NJ, Sherwood E, Harjula SKE, Hammarén MM, Ahava MJ (2013). An adult zebrafish model for preclinical tuberculosis vaccine development. Vaccine..

[68] Palha N, Guivel-Benhassine F, Briolat V, Lutfalla G, Sourisseau M, Ellett F (2013). Real-time whole-body visualization of chikungunya virus infection and host interferon response in zebrafish. PLoS Pathog.

[69] Passoni G, Langevin C, Palha N, Mounce BC, Briolat V, Affaticati P (2017). Imaging of viral neuroinvasion in the zebrafish reveals that Sindbis and chikungunya viruses favour different entry routes. Dis Model Mech.

[70] Patterson H, Saralahti A, Parikka M, Dramsi S, Trieu-Cuot P, Poyart C (2012). Adult zebrafish model of bacterial meningitis in *Streptococcus agalactiae* infection. Dev Comp Immunol.

[71] Phalipon A, Mulard LA, Sansonetti PJ (2008). Vaccination against shigellosis: is it the path that is difficult or is it the difficult that is the path?. Microbes Infect.

[72] Ravindran C, Varatharajan GR, Raju R, Vasudevan L, Anantha SR (2015). Infection and pathogenecity of *Myroides odoratimimus* (NIOCR-12) isolated from the gut of grey mullet (*Mugil cephalus* (Linnaeus, 1758)). Microb Pathog.

[73] Risalde MA, López V, Contreras M, Mateos-Hernández L, Gortázar C, de la Fuente J (2018). Control of mycobacteriosis in zebrafish (*Danio rerio*) mucosally vaccinated with heat-inactivated *Mycobacterium bovis*. Vaccine..

[74] Rocker AJ, Weiss AR, Lam JS, Van Raay TJ, Khursigara CM (2015). Visualizing and quantifying *Pseudomonas aeruginosa* infection in the hindbrain ventricle of zebrafish using confocal laser scanning microscopy. J Microbiol Methods.

[75] Salgado-Pabón W, Konradt C, Sansonetti PJ, Phalipon A (2014). New insights into the crosstalk between Shigella and T lymphocytes. Trends Microbiol.

[76] Saralahti A (2019). A zebrafish model for host-pathogen interactions in streptococcal infections.

[77] Saralahti A, Rämet M (2015). Zebrafish and streptococcal infections. Scand J Immunol.

[78] Scapigliati G, Fausto AM, Picchietti S (2018). Fish lymphocytes: an evolutionary equivalent of mammalian innate-like lymphocytes?. Front Immunol.

[79] Shahin K, Shinn AP, Metselaar M, Ramirez-Paredes JG, Monaghan SJ, Thompson KD (2019). Efficacy of an inactivated whole-cell injection vaccine for nile tilapia, *Oreochromis niloticus* (L), against multiple isolates of *Francisella noatunensis* subsp. orientalis from diverse geographical regions. Fish Shellfish Immunol.

[80] Shan Y, Fang C, Cheng C, Wang Y, Peng J, Fang W (2015). Immersion infection of germ-free zebrafish with *Listeria monocytogenes* induces transient expression of innate immune response genes. Front Microbiol.

[81] Shan Y, Zhang Y, Zhuo X, Li X, Peng J, Fang W (2016). Matrix metalloproteinase-9 plays a role in protecting zebrafish from lethal infection with *Listeria monocytogenes* by enhancing macrophage migration. Fish Shellfish Immunol.

[82] Sullivan C, Matty MA, Jurczyszak D, Gabor KA, Millard PJ, Tobin DM, Kim CH. Infectious disease models in zebrafish. In: Methods in cell biology, vol. 138: Academic; 2017. p. 101–36. 10.1016/bs.mcb.2016.10.005. 10.1016/bs.mcb.2016.10.005 28129840

[83] Tandberg J, Oliver C, Lagos L, Gaarder M, Yáñez AJ, Ropstad E, Winther-Larsen HC (2017). Membrane vesicles from Piscirickettsia salmonis induce protective immunity and reduce development of salmonid rickettsial septicemia in an adult zebrafish model. Fish Shellfish Immu..

[84] Tenor JL, Oehlers SH, Yang JL, Tobin DM, Perfect JR (2015). Live imaging of host-parasite interactions in a zebrafish infection model reveals cryptococcal determinants of virulence and central nervous system invasion. MBio..

[85] Toh MC, Goodyear M, Daigneault M, Allen-Vercoe E, Van Raay TJ (2013). Colonizing the embryonic zebrafish gut with anaerobic bacteria derived from the human gastrointestinal tract. Zebrafish..

[86] Torraca V, Gomes MC, Sarris M, Mostowy S (2019). Meeting report: zebrafish infection and immunity 2019. Lab Anim.

[87] Tsang M (2010). Zebrafish: a tool for chemical screens. Birth Defects Res C Embryo Today.

[88] Van Dycke J, Ny A, Conceição-Neto N, Maes J, Hosmillo M, Cuvry A (2019). A robust human norovirus replication model in zebrafish larvae. PLoS Pathog.

[89] Varela M, Figueras A, Novoa B (2017). Modelling viral infections using zebrafish: innate immune response and antiviral research. Antivir Res.

[90] Voelz K, Gratacap RL, Wheeler RT (2015). A zebrafish larval model reveals early tissue-specific innate immune responses to *Mucor circinelloides*. Dis Model Mech.

[91] Wang Y, Ren Z, Fu L, Su X (2016). Two highly adhesive lactic acid bacteria strains are protective in zebrafish infected with *Aeromonas hydrophila* by evocation of gut mucosal immunity. J Appl Microbiol.

[92] White RM, Sessa A, Burke C, Bowman T, LeBlanc J, Ceol C (2008). Transparent adult zebrafish as a tool for in vivo transplantation analysis. Cell Stem Cell.

[93] WHO. 2016. Human challenge trials for vaccine development: regulatory considerations. Expert Committee on Biological Standardisation. October. Geneva. https://www.who.int/biologicals/expert_committee/Human_challenge_Trials_IK_final.pdf. Accessed 27 Feb 2020.

[94] WHO (2019). Health topics: vaccines.

[95] Wiles TJ, Norton JP, Smith SN, Lewis AJ, Mobley HL, Casjens SR (2013). A phyletically rare gene promotes the niche-specific fitness of an *E. coli* pathogen during bacteremia. PLoS Pathog.

[96] Wu XM, Cao L, Hu YW, Chang MX (2019). Transcriptomic characterization of adult zebrafish infected with *Streptococcus agalactiae*. Fish Shellfish Immunol.

[97] Ye J, Kaattari IM, Kaattari SL (2011). The differential dynamics of antibody subpopulation expression during affinity maturation in a teleost. Fish Shellfish Immunol.

[98] Ye N, Wu H, Zhang Y (2016). Maternal transfer and protection role in zebrafish (*Danio rerio*) offspring following vaccination of the brood stock with a live attenuated *Vibrio anguillarum* vaccine. Aquac Res.

[99] Zhang C, Willett C, Fremgen T (2003). Zebrafish: an animal model for toxicological studies. Curr Protoc Toxicol.

[100] Zhang D, Thongda W, Li C, Zhao H, Beck BH, Mohammed H (2017). More than just antibodies: protective mechanisms of a mucosal vaccine against fish pathogen *Flavobacterium columnare*. Fish Shellfish Immunol.

[101] Zhang H, Shen B, Wu H, Gao L, Liu Q, Wang Q (2014). Th17-like immune response in fish mucosal tissues after administration of live attenuated *Vibrio anguillarum* via different vaccination routes. Fish Shellfish Immunol.

